# Study of the Stiffness of the Bitumen Emulsion Based Cold Recycling Mixes for Road Base Courses

**DOI:** 10.3390/ma13235473

**Published:** 2020-12-01

**Authors:** Katarzyna Konieczna, Piotr Pokorski, Wojciech Sorociak, Piotr Radziszewski, Dawid Żymełka, Jan Bolesław Król

**Affiliations:** 1 Faculty of Civil Engineering, Warsaw University of Technology, Al. Armii Ludowej 16, 00-637 Warsaw, Poland; p.pokorski@il.pw.edu.pl (P.P.); p.radziszewski@il.pw.edu.pl (P.R.); j.krol@il.pw.edu.pl (J.B.K.); 2 Faculty of Civil Engineering, Silesian University of Technology, ul. Akademicka 5, 44-100 Gliwice, Poland; wojciech.sorociak@eurovia.pl; 3 Eurovia Polska S.A., Bielany Wrocławskie, ul. Szwedzka 5, 55-040 Kobierzyce, Poland; dawid.zymelka@eurovia.pl

**Keywords:** cold recycling mixtures, bitumen-stabilized materials, ITSM, mechanistic-empirical pavement design, bitumen emulsion

## Abstract

The benefits of the use of cold recycling mixtures (CRMs) in pavement rehabilitation are associated with both the reduction of natural resource consumption by replacing them with recycled materials and the reduction of energy consumption during their production and paving. The evolution of the stiffness of CRMs in road construction and the fatigue life of pavements with CRM base layers are still being investigated. In this paper, CRMs with 1% cement content, called bitumen-stabilized materials with bitumen emulsion (BSM-Es), were examined. Mixtures that were differentiated in terms of Reclaimed Asphalt Pavement (RAP) content, as well as the amount and type of bitumen emulsions, were subjected to indirect tensile stiffness modulus (ITSM) tests at 5 °C, 13 °C, and 20 °C. The thermal sensitivities of the BSM-E mixtures were analyzed. BSM-E mixture stiffness modulus levels at various temperatures were determined using a statistical approach. On the basis of the results obtained, a discussion on the mechanistic-empirical design of flexible pavements with BSM-E base layers is presented. The potential benefits of using BSM-E materials in road construction in certain aspects of pavement life are indicated.

## 1. Introduction

In the era of limited availability of natural resources and growing awareness of environmental issues, cold recycling mixtures (CRMs) are gaining popularity in terms of pavement base layer construction. The main advantages of this technology are the reduction of costs, energy consumption, and greenhouse gas emissions in comparison with conventional hot mix asphalt (HMA) production [1,2].

The properties of CRMs depend on the qualitative and quantitative selection of their components, which are Reclaimed Asphalt Pavement (RAP), virgin aggregates, bitumen emulsion or foamed bitumen, water, and active filler. Depending on the applied combination of binding agents, various types of cold recycling mixtures can be distinguished, such as cement-treated materials (CTMs) with cement as the only binding agent, cement–bitumen-treated materials (CBTMs) containing bitumen emulsion or foamed bitumen with a ratio of residual bitumen (B) to cement (C) less than or equal to one (B/C ≤ 1), and bitumen-stabilized materials (BSMs) with a residual bitumen content of approximately 3% and a maximum amount of active filler (e.g., cement) equal to 1% (B/C ≥ 1) [3,4].

While CTMs are considered to be bound materials with high stiffness, rigidity, and brittleness [5,6,7], bitumen-stabilized materials (BSMs), due to a relatively high bitumen content and reduced amount of cement, are characterized by flexibility, lower stiffness, and a lower tendency to experience shrinkage cracking. This is due to the fact that the binder in BSMs is selectively dispersed among the fine aggregate particles, creating local bonds between the coarse aggregate skeleton [8,9]. Due to this phenomenon, mixtures are characterized by the combination of the mechanical properties of unbound (stress-dependent behavior) and viscoelastic materials (time and temperature-dependent behavior) [10,11,12]. The role of an active filler in BSMs with bitumen emulsion (BSM-Es) is mostly related to the acceleration of the bitumen emulsion breaking process and decreasing the moisture susceptibility of the material [8,9,13]. The mode of failure for BSMs is considered to be permanent deformation [3,8,9].

The quantitative and qualitative selection of ingredients plays an extremely important role in developing the mechanical and durability properties of CBTM and BSM mixtures, as reported by many researchers [14,15,16,17]. According to the extensive research of Dołzycki and Jaskula [18], pavement sections used in recent decades in Poland which were made with base layers with CBTM mixtures (referred to in the paper as mineral-cement-emulsion (MCE) mixtures) developed transverse reflective cracking. The main reasons for this phenomenon were considered to be excessively high cement contents (above 2.5%) in the CBTM (MCE) mixtures and, simultaneously, insufficient bitumen emulsion contents (often below 3% of the mixture mass), which resulted in an overly stiff mixture prone to cracking. This can lead to the conclusion that CBTM base layers with high cement contents (2.5% and more) may exhibit a two-phase behavior: a pre-cracked phase with higher initial stiffness and a post-cracked phase with a significantly lower stiffness of the material.

The aspects of fatigue behavior, as well as the cracking resistance of cold recycling mixtures, are considered complex and have not been fully investigated [19,20,21,22]. As BSMs cannot be characterized and modelled as purely viscoelastic nor as granular materials [11,12,23], the behavior of the BSM base layers in the pavement structure is still under discussion. Two leading hypotheses that present different approaches regarding the evolution of the material’s stiffness in construction have been developed so far. The first hypothesis, developed by Liebenberg and Visser [24,25], is based on the structural design of semi-rigid pavements. It assumes two phases of BSM performance: an effective fatigue phase, in which a high initial stiffness gradually diminishes due to fatigue, and an equivalent granular phase when the BSM layer is cracked and behaves similarly to an unbound granular material, although with a slightly greater stiffness modulus than the parent material (terminal stiffness).

According to the second hypothesis, presented by Ebels [13], the service life of BSMs consists of a curing phase, with an increase in initial stiffness resulting from moisture reduction and densification of the BSM layer, and a stiffness reduction phase, when the BSMs demonstrate the quasi-viscoelastic behavior with temperature, time, and load frequency dependence characteristics for hot mix asphalt (HMA). The second concept of stiffness development is likely to be more appropriate to the service life of BSMs, according to indirect tensile tests (ITTs) and falling weight deflectometer (FWD) measurement results in the framework of internationally conducted pilot projects [26].

The research presented in this paper consists of laboratory testing and discussion on the mechanistic-empirical design of flexible pavements with base layers consisting of bitumen-stabilized materials with bitumen emulsion (BSM-E). The experimental program focuses on the determination of the stiffness modulus (ITSM) of BSM-E mixtures at 5 °C, 13 °C, and 20 °C. Based on the obtained results, the thermal sensitivity and stiffness levels for BSM-E mixtures at various temperatures were characterized. In the second part of the paper, the fatigue life of flexible pavement with BSM-E base layers was determined according to the AASHTO 2004 pavement design procedure. An assumption was made that the BSM-E mixture retained its homogeneity in its entire service life (i.e., no brittle fracture occurred). The calculations performed for the pavement structures with BSM-E base layers were compared to those performed for reference structures with CBTM base layers used on site in Poland.

## 2. Materials and Methods

### 2.1. Materials and BSM-E Mix Design

Eight types of BSM-E mixtures were designed. Differentiation of the mixtures was made in terms of the amount of RAP (70% and 50% of the mineral mixture mass), as well as the amount and type of bitumen emulsion used. Additionally, one reference CBTM mixture with 50% RAP, 3.0% cement and 3.0% bitumen emulsion addition was designed.

**Figure 1:**
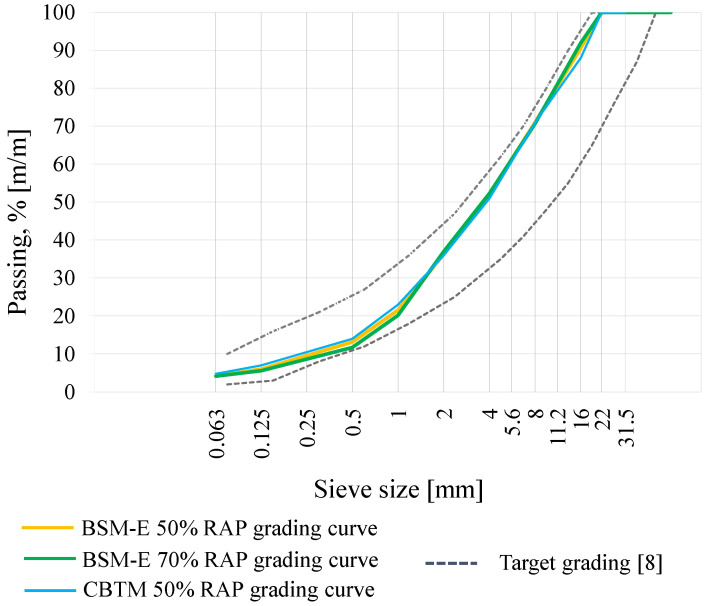
Bitumen-stabilized materials with bitumen emulsion (BSM-E) and cement–bitumen-treated material (CBTM) grading curves with target grading [8].

Grading curves of the BSM-E mixtures were designed using 50% and 70% RAP 0/31.5 mm, derived from milling and wearing and base asphalt courses originally containing dolomite 0/11 and basalt 11/16 aggregates. The average bitumen content in the RAP was determined to be 5.0% by its aggregate weight (PN-EN 12697-1). In order to meet the grading criteria presented in the international standards for BSMs [8], virgin aggregates, such as continuously graded basalt aggregate 0/31.5 mm and fine basalt aggregate 0/2 mm, were added to the mixture. The mixing proportions of the RAP and virgin aggregates were set to obtain a similar shape for the grading curves of the BSM-E and CBTM mixtures, as shown in Figure 1.

Two types of slow-setting cationic bitumen emulsions with 60% residual bitumen content (type 1: E1, with a bitumen emulsion produced on the base of 50/70 penetration grade bitumen, and type 2: E2, with a bitumen emulsion of C60B10 ZM/R with 70/100 penetration grade bitumen) and the cement type CEM II/B-S 32.5 R (EN 197-1) were used to prepare the BSM-E mixtures. The amount of cementitious binder was set to a constant 1% of the mixture mass. The optimum moisture content (OMC) for mixtures was determined in accordance with the modified Proctor moisture–density relationship test procedure (PN–EN 13286-2). The OMC was set to 6.8% for BSM-E mixtures containing 70% RAP and 7.6% for BSM-E mixtures containing 50% RAP. The OMC of the CBTM mixture was set to 5.0%. The mixture compositions of the BSM-E mixtures and CBTM mixture are listed in Table 1.

### 2.2. Specimen Preparation and Curing

The BSM-E and CBTM mixtures were mixed and compacted at ambient temperatures (25 °C). The test specimens, with a height of 63.5 mm ± 3.5 mm and a diameter of 101.6 mm, were compacted using a Marshall hammer by applying 75 blows per side, according to national requirements [27]. Perforated molds with 24 holes 2 mm in diameter were used in order to facilitate the drainage of water during the compaction process. Once compacted, specimens were left in moulds for 24 h in order to develop sufficient strength before demoulding. After that, for the BSM-E specimens, the accelerated curing protocol was adopted [8]. The specimens were placed in a forced-air oven at 40 °C and cured for 72 h. After the curing process, specimens were cooled at ambient temperature (25 °C). For the CBTM specimens, due to the higher content of cement, the curing conditions presented in national requirements were applied (28 days, 25 °C, and air humidity 40–70%) [27].

Bulk densities of the BSM-E specimens ranged from 2.092 Mg/m^3^ to 2.138 Mg/m^3^ for mixtures containing 70% RAP in the mineral mixture and from 2.144 Mg/m^3^ to 2.160 Mg/m^3^ for mixtures containing 50% RAP. The average bulk density of the CBTM mixture specimens was 2.207 Mg/m^3^.

### 2.3. Indirect Tensile Stiffness Modulus (ITSM) Testing

A UTM-25 universal testing machine was used to carry out stiffness modulus tests (ITSM) at 5 °C, 13 °C, and 20 °C in an indirect tensile configuration (IT-CY) according to PN-EN 12697-26. A target deformation value of 5 μm was selected in accordance with national instructions [27] in order to avoid premature brittle failure by the specimens during the early stages of the test. Measurements were conducted on at least 3 specimens (on average, 6 samples for each mixture) along two perpendicular diameters for each specimen, and the average ITSM values for each mixture type and each testing temperature were calculated.

### 2.4. Methodology of Flexible Pavement Design with a Cold Recycling Mix (CRM) Base Layer

Design of the flexible pavement with a BSM-E base layer was carried out according to fixed pavement structures presented in the national design and construction guide *The Catalogue of Typical Flexible and Semi-rigid Pavements* [28]. The mechanistic-empirical method was used based on the following criteria: bottom-up fatigue cracking of the bituminous layers and permanent deformation (AASHTO 2004 pavement design procedure). In order to calculate the stress and strain state in the pavement structures, the theory of elastic layered half space was applied. Calculations were conducted using BISAR 3.0 software under the assumption that the single axis traffic load was represented by a circular contact area with *P* = 50 kN load and *q* = 850 kPa contact pressure.

The comparison of the fatigue lives was carried out between flexible pavement structures with base layers constructed with BSM-E and CBTM mixtures. Both the BSM-E and CBTM layers were considered as flexible. In accordance with the national design guide recommendations [28], the thickness (*h*_i_), Poisson’s ratio (*ν*_i_), and modulus of elasticity (stiffness) (*E*_i_) at the equivalent temperature of 13 °C were specified for each pavement layer. The stiffness modulus of the traditionally used CBTM mixtures was set to 1500 MPa, and the Poisson’s ratio was set to 0.30. The value of 1500 MPa was the stiffness modulus value, which is recommended for calculations in the Polish guidelines for CBTM mixtures [28]. There is no recommended stiffness modulus value separately established for BSM-E mixtures. Due to the variety of stiffness modulus values obtained both in the laboratory and during in situ testing for the CRMs, the assumption of a single stiffness modulus value of 1500 MPa for a wide range of cold recycling mixtures is still being discussed. As stated in the paper by Dolzycki and Jaskula [18], although the 1500 MPa value was lower than the values obtained during laboratory and in situ testing of the CBTM mixtures (referred to in the paper as mineral-cement-emulsion (MCE) mixtures), it was supposed to represent the inhomogeneous material after a certain period of performance, namely when the first distress and micro-cracks began to occur. The BSM-E stiffness modulus for pavement design purposes was calculated using a statistical approach on the basis of the results obtained in this research.

## 3. Results and Discussion

### 3.1. Indirect Tensile Stiffness Modulus (ITSM)

**Figure 2:**
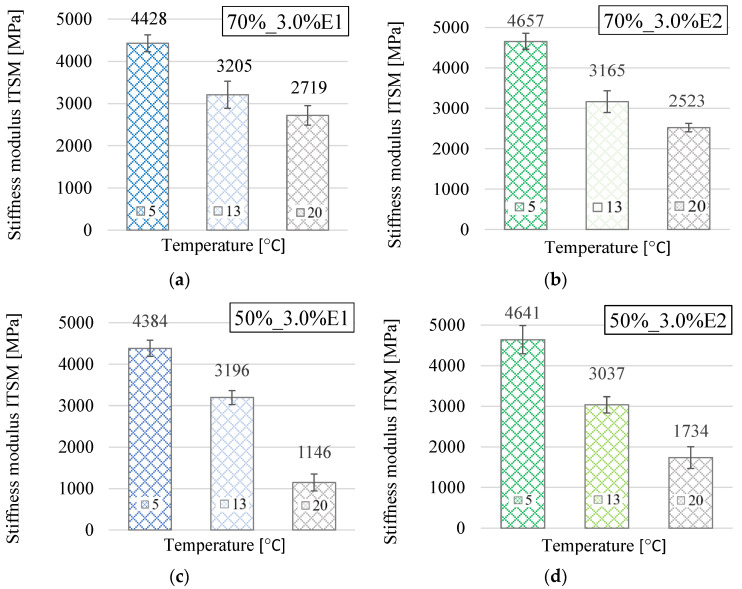

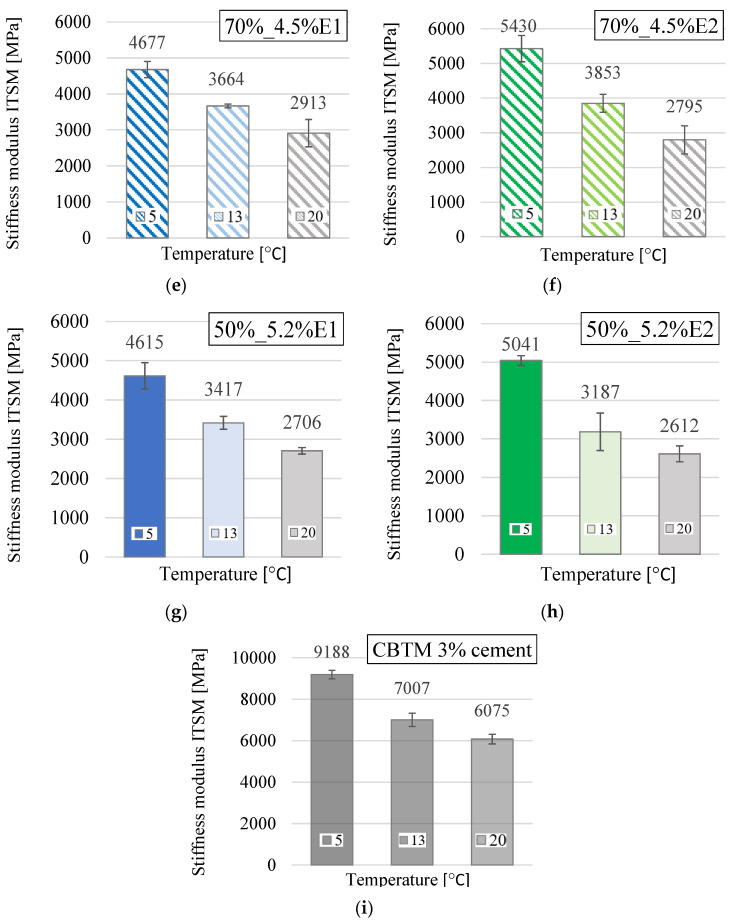
ITSM test results for BSM-E and CBTM mixtures at 5 °C, 13 °C, and 20 °C. (**a**) BSM-E 70%_3.0%E1, (**b**) BSM-E 70%_3.0%E2, (**c**) BSM-E 50%_3.0%E1, (**d**) BSM-E 50%_3.0%E2, (**e**) BSM-E 70%_4.5%E1, (**f**) BSM-E 70%_4.5%E2, (**g**) BSM-E 50%_5.2%E1, (**h**) BSM-E 50%_5.2%E2, and (**i**) CBTM with 3% cement content.

The results of the ITSM tests at 5 °C, 13 °C, and 20 °C, presented in Figure 2a–i, show that the ITSM values of the BSM-E and CBTM mixtures decreased with the increase of the testing temperature. The average stiffness moduli of all BSM-E mixtures ranged from 4382 MPa to 5215 MPa at a temperature of 5 °C, from 3037 MPa to 3721 MPa at 13 °C, and from 1146 MPa to 2913 MPa at 20 °C. While comparing these values with the values obtained for the CBTM mixture containing 3% cement, it could be stated that the average stiffness of the BSM-E mixtures was approximately 50–60% lower.

To evaluate statistically significant differences in the BSM-E stiffness modulus values, one-way analysis of variance (ANOVA) using Statgraphics Centurion 18.1.06 software was performed. By means of the F-test, it was concluded that there was a statistically significant difference between the ITSM mean values at the 5% significance level for each testing temperature. Based on the stiffness modulus values presented in Figure 2a–i, it can be observed that the BSM-E mixtures containing 50% RAP were charaterized by lower ITSM values than the corresponding BSM-E mixtures with a higher RAP content. However, the multiple range tests did not show statistically significant differences between corresponding mixtures with different RAP percentages.

In the case of the BSM-E mixtures with 50% RAP and 3.0% bitumen emulsion content, a significant decrease in the value of the ITSM stiffness modulus at 20 °C was observed. Such a tendency was not observed for the BSM-E mixtures with 70% RAP content.

It can be assumed that this reduction of the stiffness is related to the loss of cohesion of the mixtures with low contents of bitumen emulsion (1.8% residual binder) and relatively low contents of RAP. Although RAP has so far been mainly treated as a black rock in cold recycling mixtures, it is not without significance that the old binder surrounding the RAP particles could still take part in binding the aggregates together during compaction [29]. Ebels [13] reported that the RAP particles present in cold recycling mixtures were characterized by a lower absorption rate of fresh bitumen compared with virgin aggregates, which resulted in a greater affinity between the fresh bitumen and the RAP particles.

### 3.2. Temperature Dependency

**Figure 3:**
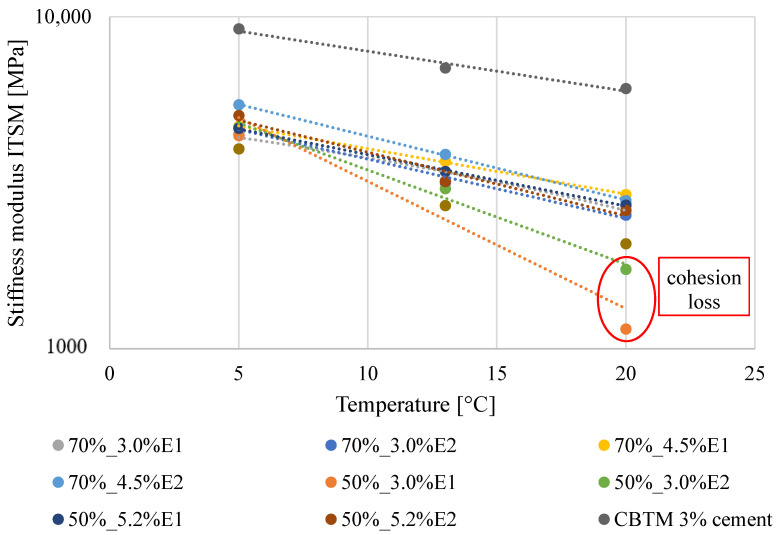
ITSM values as a function of the testing temperature.

As mentioned in Section 3.1, the obtained ITSM test results confirmed the temperature dependency of the BSM-E mixture, displaying a decrease in ITSM values with the increase in the testing temperature (Figure 3). The stiffness and temperature relationships were based on the average values at three different temperatures. The error bars are not shown in Figure 3 (given in Figure 2), taking into account the clarity of the presented relationships.

In order to quantify the temperature dependency of the BSM-E mixtures, the following equation was proposed:

log *S* = −*A*_*ITSM*_ · *t* + *B_ITSM_* (1)

where *S* is the ITSM stiffness modulus at temperature *t* and *A_ITSM_* and *B_ITSM_* are parameters depending on the BSM-E mixture characteristics. The fitting parameters of the regression curve for the BSM-E mixtures are presented in Table 2.

Considering the R^2^ values presented in Table 2, it can be stated that the applied analytical model allowed a good fitting with the ITSM results (R^2^ = 0.9659 – 0.9983). The 50%_3.0%E1 mixture was the exception, with an R^2^ value of 0.8918. Regression curves for the BSM-E mixtures with 50% RAP and 3.0% bitumen emulsion signifficantly differed from those obtained for other mixtures as a result of a significant drop in the stiffness modulus value at a temperature of 20 °C (cohesion loss). The regression curve slope values (represented by *A_ITSM_*) described the gradient of stiffness modulus change as a function of the testing temperature; the higher the slope (*A_ITSM_*) value, the more temperature-sensitive the BSM-E mixture was. Based on the calculated *A_ITSM_* values, it can be stated that all the BSM-E mixtures containing type E2 bitumen emulsion with a 70/100 binder (except for 50%_3,0%E2) were characterized by a higher temperature dependency than corresponding mixtures containing type E1 bitumen emulsion with a 50/70 penetration grade binder. In comparison with the CBTM mixture, the BSM-E mixtures presented higher *A_ITSM_* values, which stood for a higher sensitivity to changes in the test temperature.

### 3.3. Statistical Analysis of the BSM-E Stiffness Level

Statistical analysis of the results was performed to evaluate the level of BSM stiffness at the three test temperatures (5 °C, 13 °C, and 20 °C). The ITSM test results, divided into three groups, were evaluated using statistical data analysis tools (Statgraphics Centurion 18.1.06 software). Probability distribution fitting was conducted, and box plots were created to illustrate the dispersion of the obtained ITSM values in each group. Statistical parameters of the tested statistical samples are presented in Table 3.

Based on the application of the Shapiro–Wilk test, it was concluded that at the adopted level of significance α = 0.05, there were no grounds for rejecting the hypothesis that the analyzed ITSM results in all three samples came from the normal distribution (Shapiro–Wilk test *p*-value > α = 0.05). The general stiffness levels of the BSM-E mixtures were defined as stiffness modulus values *S_temp_*, for which the probability of getting a lower ITSM value was approximately equal to 5% (P (X < *S_temp_*) ≅ 0.05).

**Figure 4:**
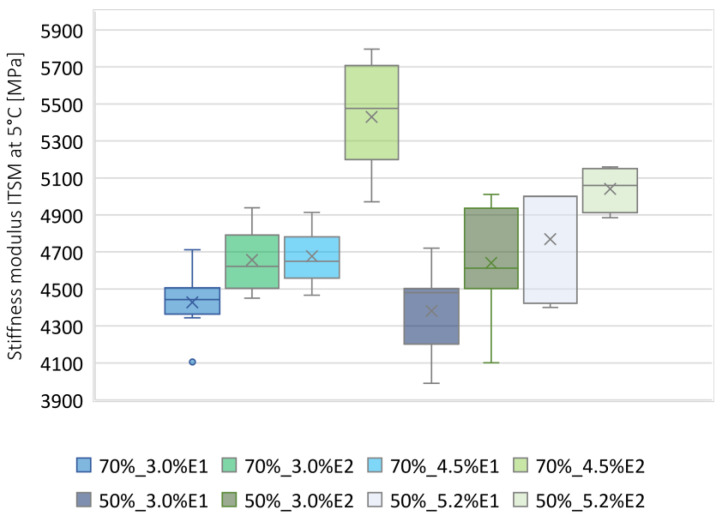
Box plot of ITSM values at 5 °C.

For the test temperature of 5 °C, the average ITSM value was calculated as 4678 MPa, while the standard deviation of the results was equal to 406 MPa. As shown in Figure 4, the BSM-E mixture 70%_4.5%E2 was characterized by the highest average stiffness modulus at 5 °C. Individual values for the other mixtures were in the range of 3990–5159 MPa.

**Figure 5:**
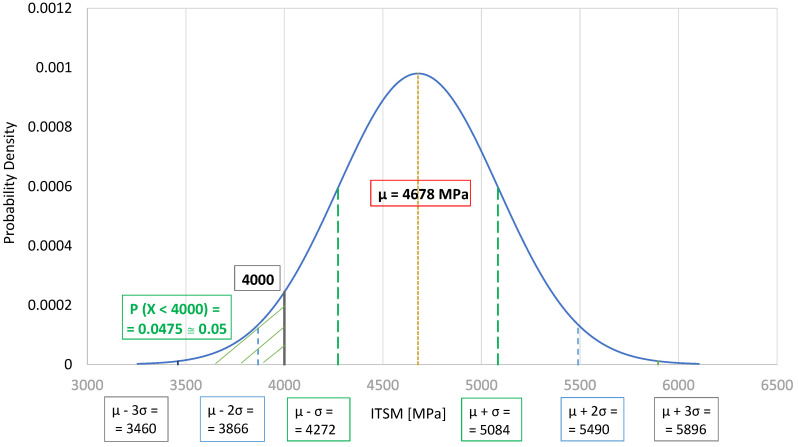
Probability density function of a normal distribution N (4678; 406) for ITSM values at 5 °C.

On the basis of the conclusions made, the probability density function of a normal distribution with a mean value *μ* and a standard deviation *σ*: *N* (*μ* = 4678, *σ* = 406) was created (Figure 5).

Based on the areas limited by subsequent standard deviation values that had been marked in the Gaussian curve, the value of 4000 MPa was defined as the BSM-E stiffness level at 5 °C.

**Figure 6:**
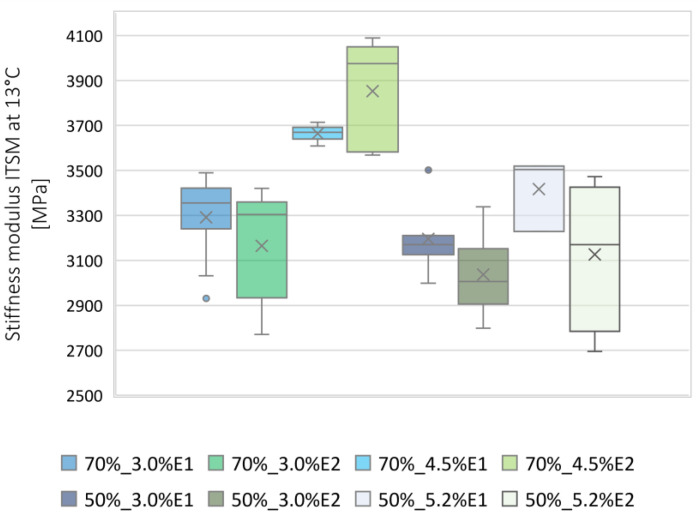
Box plot of ITSM values at 13 °C.

**Figure 7:**
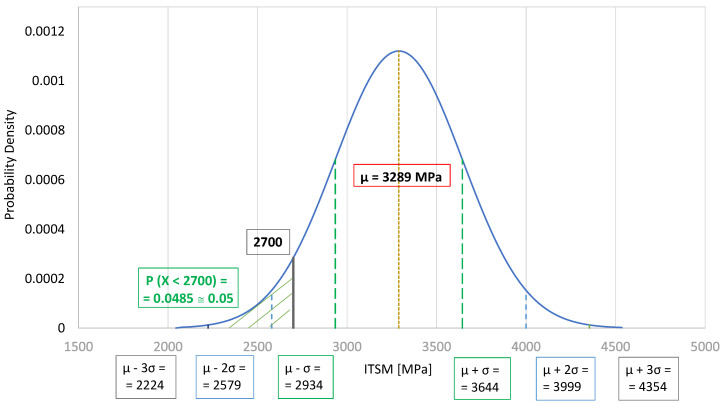
Probability density function of a normal distribution N (3289; 355) for ITSM values at 13 °C.

For the test temperature of 13 °C, the average ITSM value was calculated to be 3289 MPa, while the standard deviation of the results was equal to 355 MPa. Individual ITSM values for the BSM-E mixtures were in the range of 2695–4089 MPa, with the 70%_4.5%E2 mixture characterized by the highest average stiffness modulus (Figure 6). The probability density function of a normal distribution N (3289; 355) for the ITSM values is presented in Figure 7. The value of 2700 MPa was defined as the BSM-E stiffness level at 13 °C. According to national requirements [28], the stiffness modulus value at 13 °C is used as a parameter for flexible pavement design.

**Figure 8:**
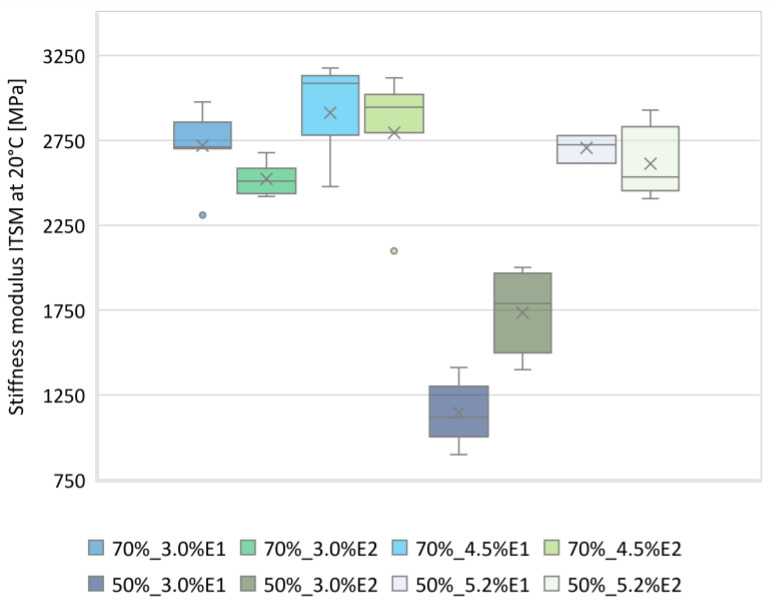
Box plot of ITSM values at 20 °C.

**Figure 9:**
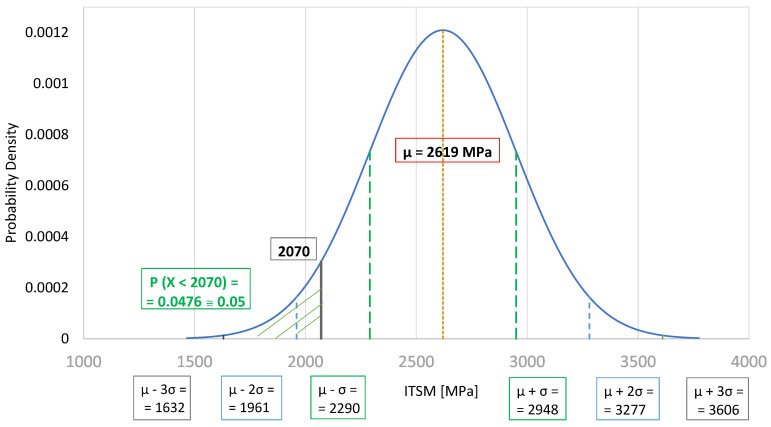
Probability density function of a normal distribution N (2619; 329) for ITSM values at 20 °C.

As presented in Figure 8, at a temperature of 20 °C, the ITSM values of the BSM-E mixtures with 50% RAP and 3.0% bitumen emulsion were significantly lower than the values obtained for other mixtures. Therefore, the stiffness moduli of those mixtures were considered as outliers and were not taken into account in the statistical analysis. The average ITSM value, based on the rest of the results, was calculated as 2619 MPa, while the standard deviation of the results was equal to 329 MPa. The probability density function of a normal distribution N (2619; 329) for the ITSM values at 20 °C is presented in Figure 9. The value of 2070 MPa was defined as the stiffness level of the BSM-E mixtures at the temperature of 20 °C.

**Figure 10:**
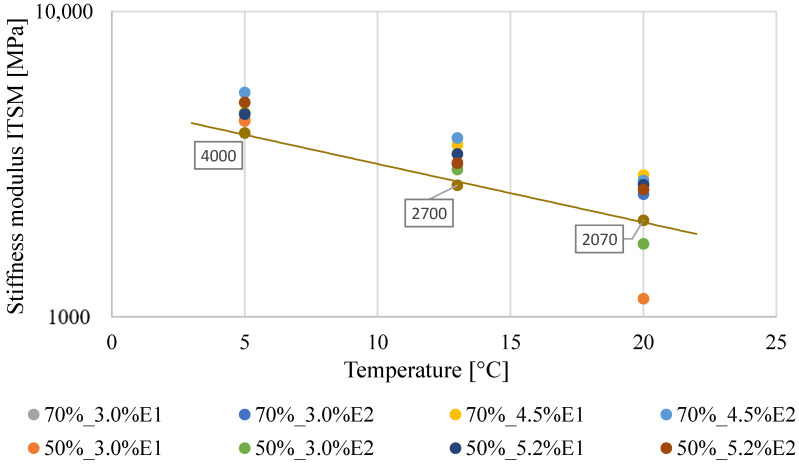
Regression curve for the specified BSM-E stiffness values at 5 °C, 13 °C, and 20 °C.

Figure 10 presents the regression curve for the calculated BSM-E stiffness modulus levels (*S_temp_*) and single points that represent the average ITSM values for the designed BSM-E mixtures at temperatures of 5 °C, 13 °C, and 20 °C. It can be seen that the specified *S_temp_* values were lower than the laboratory-tested average ITSM values for the BSM-E mixtures (except for the results obtained for the BSM-E mixtures with 50% RAP and 3.0% bitumen emulsion at 20 °C).

## 4. Analysis of Flexible Pavement Structures with BSM-E Base Layers

Table 4 presents the results of the pavement life analysis conducted for the reference structure, consisting of flexible pavement with a base layer made of a CBTM mixture traditionally used in Poland and a pavement structure with a BSM-E base layer. The Polish traffic categories KR1–KR2 are considered to be light traffic, whereas categories KR3–KR4 stand for medium traffic.

It can be observed that for both variants of the pavement structure, pavement life was determined by the permanent (structural) deformation criterion. The results of calculations of the flexible pavement structure with a base layer of a BSM-E mixture indicated a significant reserve of the required pavement life (over 200%) for all traffic categories. The obtained numbers of equivalent single axle loads (100 kN) to failure for pavements with a BSM-E layer were approximately three to five times greater than those calculated for pavement structures with a CBTM mixture characterized by a stiffness modulus equal to 1500 MPa. Such a significant overdesign of the pavement structures with BSM-E encouraged authors to propose alternative design solutions. For technological reasons, in the case of traffic categories KR1 and KR2, the thickness of the asphalt layer packet had not been changed. The analysis of the fatigue life of category KR1–KR2 pavement structures led to a conclusion that the use of BSM-E base layers could bring significant benefits in terms of the extended service life and reduction of possible maintenance and rehabilitation cost of local roads with light traffic loading. Alternative design solutions for flexible pavements with BSM-E base layers in medium traffic categories (KR3–KR4) are shown in Table 5.

The main goal of creating pavement structure alternatives was to obtain similar values of pavement life for both basic and alternative design solutions while ensuring construction criteria was followed. In that case, the incorporation of the BSM-E layer allowed authors to propose the reduction of the suggested thicknesses of asphalt layer packets by 2 cm for KR3 as well as for the KR4 traffic category. It can be observed that the values of the tensile stresses at the bottom of the asphalt layers decreased by approximately 40% for the KR3 traffic category and 20% for the KR4 traffic category, in comparison with the basic pavement solutions with CBTM mixtures. The potential possibility of reducing the thickness of asphalt layer packets in the case of medium traffic category roads without negatively affecting pavement life has unquestionable benefits in terms of construction material savings, as well as construction, maintenance, and rehabilitation cost reduction.

In order to introduce alternative solutions for high traffic categories (KR5–KR7) that are not included in the design guide [28], the results of laboratory testing and material behavior assumptions should be confirmed beforehand and complemented by a real scale analysis. For instance, falling weight deflectometer (FWD) tests are advised to be carried out in certain exploitation time intervals in order to analyze the responses of pavements subjected to high traffic loads in terms of surface deflections, strain, and stress measurements.

## 5. Conclusions

The following conclusions can be drawn, based on laboratory test results and pavement design analysis:

- Due to the reduced amount of cement, BSM-E mixtures exhibit 50–60% lower stiffness modulus values than CBTM mixtures in which a 3% cement addition is applied;
- The thermal sensitivities of the BSM-E mixtures depend on the type of bitumen emulsion used. Thermal sensitivities for the BSM-E mixtures are higher in comparison with CBTM mixtures, which may indicate that base layers made of CBTM do not exhibit the properties of flexible layers, but rather manifest behavior similar to rigid materials;
- Based on statistical analysis, assuming a 5% probability of obtaining lower stiffness modulus results, the following stiffness levels of BSM-E mixtures *S_temp_* were determined at three testing temperatures: *S_temp5_* = 4000 MPa at 5 °C, *S_temp13_* = 2700 MPa at 13 °C, and *S_temp20_* = 2070 MPa at 20 °C;
- Assuming the validity of the BSM-E behavior hypothesis that a layer’s stiffness modulus increases during the initial period of pavement exploitation, and the material does not exhibit brittle cracking and a loss of initial stiffness, the constant BSM-E stiffness modulus value of 2700 MPa can be adopted for pavement design calculations;
- The adaptation of a higher stiffness modulus for BSM-E layers (2700 MPa) in comparison with CBTM layers (1500 MPa) is possible due to the expected difference of material characteristics from rigid and more prone to shrinkage and fatigue cracking for CBTMs to flexible and more crack-resistant BSM-E layers;
- The mechanistic-empirical pavement design calculations showed that, in the case of light traffic categories KR1–KR2, the application of BSM-E mixtures in base layers may bring potential benefits in terms of extended pavement life and the durability of local roads;
- For medium traffic categories (KR3–KR4), the thicknesses of asphalt layers presented in the national requirements can be reduced without negatively affecting the pavement life, which can contribute to the reduction of construction material use.

The laboratory testing and pavement design results should be confirmed and complemented by a real scale analysis. Falling weight deflectometer (FWD) tests are recommended to be conducted in order to analyze pavement response and determine the back-calculated stiffness modulus of the BSM-E layer in certain time intervals. Such tests are crucial to verify the assumption of increased cracking resistance and flexibility of BSM-E base layers.

## Figures and Tables

**Table 1:** BSM-E and CBTM mixture compositions.

Mixture Type	Mixture Designation	Ingredient Content [%]					
		RAP	Basalt Aggregate 0/31.5 mm	Basalt Aggregate 0/2 mm	Bitumen Emulsion	Bitumen Type	Cement
BSM-E	70%_3.0%E1 70%_3.0%E2	70	–	30	3.0	50/70	1.0
						70/100	
	70%_4.5%E1 70%_4.5%E2				4.5	50/70	
						70/100	
BSM-E	50%_3.0%E1 50%_3.0%E2	50	37.5	12.5	3.0	50/70	1.0
						70/100	
	50%_5.2%E1 50%_5.2%E2				5.2	50/70	
						70/100	
CBTM	CBTM 3% Cement	50	50	–	3.0	70/100	3.0

**Table 2:** Fitting parameters for the ITSM temperature dependency, calculated in Equation (1).

ITSM	CBTM 3% Cement	70%_3.0%E1	70%_3.0%E2	50%_3.0%E1	50%_3.0%E2	70%_4.5%E1	70%_4.5%E2	50%_5.2%E1	50%_5.2%E2
*A_ITSM_* (logMPa/°C)	0.0120	0.0142	0.0178	0.0383	0.0284	0.0137	0.0193	0.0155	0.0192
*B_ITSM_* (logMPa)	4.0166	3.7086	3.7492	3.8872	3.8221	3.7395	3.8279	3.7395	3.7838
*R* ^2^	0.9809	0.9783	0.9878	0.8918	0.9861	0.9996	0.9983	0.9988	0.9659

**Table 3:** Statistical parameters of tested ITSM values samples.

Temperature (°C)	Statistical Sample Size	Average Stiffness Modulus Value (MPa)	St. Dev. (MPa)	*p*-Value (Shapiro–Wilk Test)
5	40 specimens	4678	406	0.056872
13	50 specimens	3289	355	0.777681
20	40 specimens	2619	329	0.370573

**Table 4:** Calculated pavement life of flexible pavement structures with CBTM and BSM-E base layers.

Base Layer		CBTM Mixture; E = 1500 MPa				BSM-E Mixture; E = 2700 MPa			
Traffic Category		KR1	KR2	KR3	KR4	KR1	KR2	KR3	KR4
Asphalt/CBTM or BSM-E Layers Thickness (cm)		8/15	12/15	12/20	16/20	8/15	12/15	12/20	16/20
Pavement Life (No. of Equivalent Single Axle Loads)	N1 ^1^	0.1	0.8	2.8	10.5	0.5	2.1	8.7	28.0
	N2 ^2^	17.2	9.6	12.6	17.8	252.4	60.1	87.5	86.8
	Min (N1, N2)	0.1	0.8	2.8	10.5	0.5	2.1	8.7	28.0
Required Pavement Life acc. to Traffic Category (No. Equivalent Single Axle Loads) [28]		0.03–0.09	0.09–0.50	0.5–2.5	2.5–7.4	0.03–0.09	0.09–0.50	0.5–2.5	2.5–7.4
Reserve of Pavement Life (%)		+11%	+60%	+12%	+42%	+456%	+320%	+248%	+278%

^1^ N1 = permanent deformation criterion. ^2^ N2 = fatigue cracking of bituminous layers criterion (FC = 10%).

**Table 5:** Alternative design solutions for flexible pavements with BSM-E base layers.

Traffic Category		KR3		KR4	
Pavement Design Solution		Basic Solution CBTM	Alternative Solution BSM-E	Basic Solution CBTM	Alternative Solution BSM-E
Asphalt/CBTM or BSM-E Layers Thickness (cm)		12·(4 + 8) ^i^/20	10·(4 + 6) ^i^/20	16·(4 + 5 + 7) ^ii^/20	14·(4 + 4 + 6) ^ii^/20
Tensile Strain: Bottom of Asphalt Layers (μm/m)		82.50	48.58	70.45	55.32
Compressive Strain: Top of Subgrade (μm/m)		381.8	342.9	285.5	259.50
Pavement Life (No. of Equivalent Single Axle Loads)	N1 ^1^	2.8	4.6	10.5	16.1
	N2 ^2^	12.6	118.9	17.8	48.1
	min (N1, N2)	2.8	4.6	10.5	16.1
Required Pavement Life Acc. to Traffic Category (No. of Equivalent Single Axle Loads) [28]		0.5–2.5		2.5–7.4	
Basic/Alternative Solution Pavement Life Ratio		1	1.6	1	1.5

^i^ Surface course + binder course. ^ii^ Surface course + binder course + base course. ^1^ N1 = permanent deformation criterion. ^2^ N2 = fatigue cracking of bituminous layers criterion (FC = 10%).
